# Mr. William Frank Hearnden

**Published:** 1910-07-16

**Authors:** 


### OBITUARY.

The death is announced, at Sutton, Surrey, of Mr. William Frank Hearnden, in his fifty-fourth year. He was a student at Guy's Hospital, and became M.R.C.S. in 1879, and L.S.A. the following year.

